# Changes in immunofluorescence staining during islet regeneration in a cystic fibrosis-related diabetes (CFRD) ferret model

**DOI:** 10.1080/19382014.2024.2436696

**Published:** 2024-12-06

**Authors:** Sawash M. Mohammed, Robert N. Bone, Jacqueline Del Carmen Aquino, Raghavendra G. Mirmira, Carmella Evans-Molina, Heba M. Ismail

**Affiliations:** a Center for Diabetes and Metabolic Diseases, Indiana University School of Medicine, Indianapolis, IN, USA; b Department of Anatomy, Cell Biology, and Physiology, Indiana University School of Medicine, Indianapolis, IN, USA; c Department of Pediatrics, Indiana University School of Medicine, Indianapolis, IN, USA; d Herman B. Wells Center for Pediatric Research, Indiana University School of Medicine, Indianapolis, IN, USA; e Kovler Diabetes Center and the Department of Medicine, The University of Chicago, Chicago, IL, USA

**Keywords:** Alpha and beta cells, cystic fibrosis-related diabetes, ferret model, glucagon staining, glucagon-to-insulin ratio, glycemic phases, Immunostaining, insulin staining, proinsulin staining, proinsulin-to-insulin ratio, proinsulin-to-insulin colocalization

## Abstract

**Background:**

Knockout (KO) ferrets with the cystic fibrosis transmembrane conductance regulator (CFTR) exhibit distinct phases of dysglycemia and pancreatic remodeling prior to cystic fibrosis-related diabetes (CFRD) development. Following normoglycemia during the first month of life (Phase l), hyperglycemia occurs during the subsequent 2 months (Phase Il) with decreased islet mass, followed by a period of near normoglycemia (Phase Ill) in which the islets regenerate. We aimed to characterize islet hormone expression patterns across these Phases.

**Methods:**

Immunofluorescence staining per islet area was performed to characterize islet hormone expression patterns in age matched CFTR KO and wild type (WT) ferrets, focusing on the first three phases.

**Results:**

In Phase I, insulin staining intensity was higher in CF (*p* < 0.01) than WT but decreased in Phase III (*p* < 0.0001). Glucagon was lower in CF during Phases I and increased in Phase III, while proinsulin decreased (*p* < 0.0001) Phases II and III. CF sections showed lower proinsulin-to-insulin ratio in Phase I (*p* < 0.01) and in Phase III (*p* < 0.05) compared to WT. Conversely, glucagon-to-insulin ratio was lower in CF in Phase I (*p* < 0.0001) but increased in Phase III (*p* < 0.0001). Mender’s coefficient overlap showed higher overlap of insulin over proinsulin in CF sections in Phase II (*p* < 0.001) and Phase III (*p* < 0.0001) compared to WT. Mender’s coefficient rate was higher in CF sections during Phase II (*p* < 0.001).

**Conclusion:**

CF ferret islets revealed significant immunofluorescent staining changes compared to WT during various phases of disease, providing insights into CRFD pathophysiology.

## GRAPHICAL ABSTRACT

**Figure:**
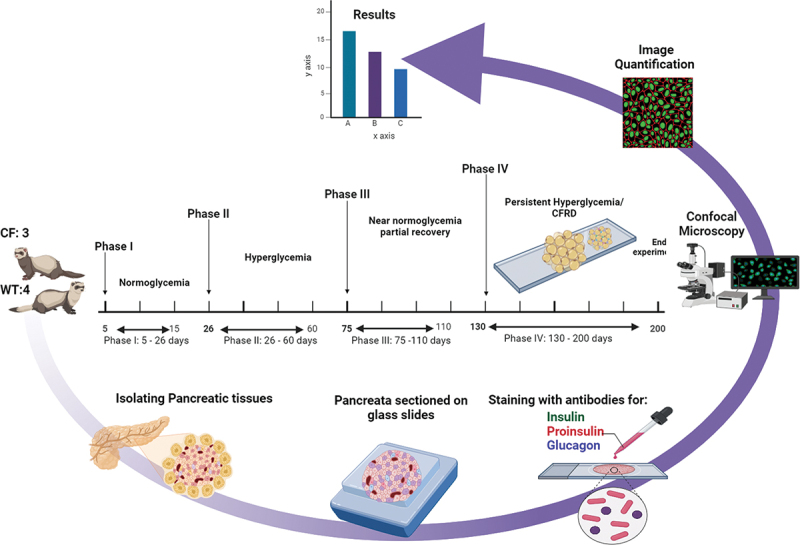

## Introduction

Cystic fibrosis-related diabetes (CFRD) occurs in 40–50% of adults with cystic fibrosis (CF) and heralds worse clinical outcomes with high morbidity and mortality^1^. CFRD has features of both type 1 diabetes (T1D) and type 2 (T2D), although deficient insulin secretion and beta-cell dysfunction prevail^2,3^. Postulated mechanisms of CFRD development include collateral damage from pancreatic exocrine destruction, inherent beta-cell defects, cystic fibrosis transmembrane regulator (CFTR) dysfunction, and incretin deficiency or unresponsiveness. However, the variable development of CFRD in CF patients has yet to be explained and, therefore, other mechanisms might be involved^2^.

It is well known that the knockout ferret for the cystic fibrosis transmembrane conductance regulator (CFTR) is a good animal model for CF^3^ that exhibits different stages of dysglycemia and pancreatic remodeling that resemble human pathophysiology^4^. The first month of life is characterized by normoglycemia/Phase I. While hyperglycemia is present (Phase II) with a decrease in the number of pancreatic islets, followed by a period of near normoglycemia (Phase III) with regeneration of the islets, and concluding with chronic hyperglycemia and CFRD (Phase IV)^2^. In children with CF, a similar trend of early life glucose intolerance, followed by normoglycemia, has been seen^5^. Considering the intriguing progression of the disease, it is important to investigate the alterations occurring in the islets during the various glycemic phases and prior to the onset of disease. Although the fluctuations in islet hormone mRNA levels have been documented throughout these phases, the changes in protein expression patterns have not been investigated^2^. The main purpose of this study is to investigate and describe these changes through immunofluorescent staining. Our study aims to understand the changes in alpha- and beta-cell immunostaining and protein expression during different phases of glycemia, underlying CFRD development.

## Materials and methods

### Pancreatic tissue sources

**Figure 1.**
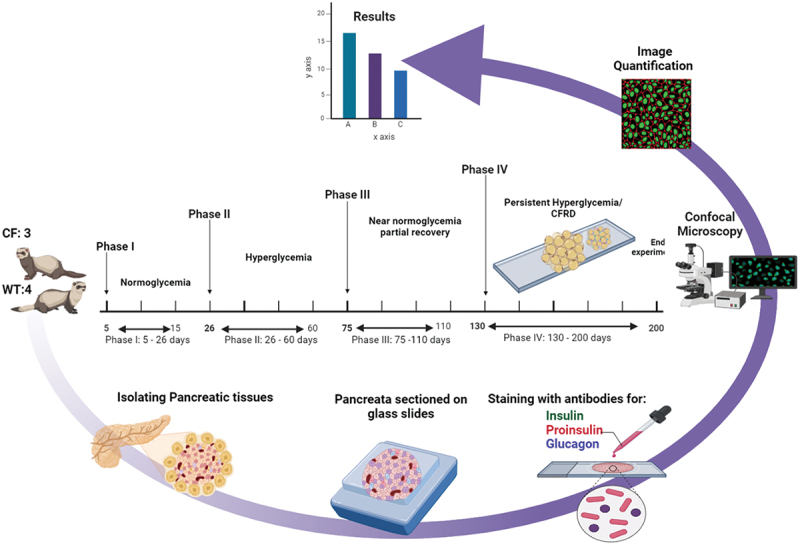
Illustration of showing the four phases of diabetes development in CF ferret models. Insulin, proinsulin, and glucagon were characterized from pancreatic sections and images compared. Phase I describes normal blood sugar levels (normoglycemia) in the first month of life, and Phase II is characterized by hyperglycemia accompanied by a decrease in islet mass. After this phase (Phase III), there is a period of near-normal blood sugar levels with regeneration of islet mass, followed by final (Phase IV) sustained hyperglycemia and cystic fibrosis-related diabetes (CFRD).

Formalin fixed paraffin embedded ferret pancreas sections were acquired from Dr. John Engelhardt’s laboratory at the University of Iowa^2^. Sections from CFTR knockout (CF) and Wild Type (WT) ferrets were used for this study. The ferrets were euthanized at various time points of each Phase (day 10 of Phase I, day 30 of Phase II, day 80 of Phase III, and day 140 of Phase IV) during the first 4 months of life to collect pancreatic tissue, according to the local guidelines of the Institutional Animal Care and Use Committee^2^. Comparative analysis was performed using samples obtained from WT ferrets as controls at the corresponding time points/stages of disease (Figure 1). Sections were compared for each phase and corresponding age for WT ferrets from the following number of animals: 2 CF Phase I and 2 WT; 3 CF Phase II and 3 WT; and 2 CF Phase III and 3 WT ferrets. The analysis included 169–259 islets from the biological replicates for each genotype in each phase. Phase IV sections were not included in this analysis as staining revealed complete replacement with fatty tissues in all sections, making a comparison invalid.

### Immunofluorescence staining

Pancreatic sections were embedded in paraffin and mounted on glass slides^6^. Briefly, the pancreas tissue was fixed overnight at 4°C in 4% paraformaldehyde. The tissue was transferred to 70% ethanol, followed by paraffin embedding and sectioning (5-μm thickness). Deparaffinization and rehydration of the pancreas sections were carried out through a series of washes in xylene twice for 5 min,100% ethanol twice for 2 min each, 95%, 90%, 80%, and 70% ethanol for 1 min each to remove xylenes and rehydrate. The sections were then washed in ddH2O twice for 2 min each. To retrieve the target antigen, slides were incubated in 1× unmasking solution (Vector Labs H-3300) for 13–15 min. Following the antigen retrieval, the slides were left uncovered at room temperature for 15 min, and then placed into the beaker under the slow stream of deionized water for 10 min. Pancreas sections were circumscribed with ImmEdge® Pen Hydrophobic Barrier (Vector Labs H-4000) and incubated for 1 h at room temperature with Animal Free Blocker (Vector SP5030-5X). Prior to applying the primary antibodies, the slides were washed twice in PBS for 5 min each. Primary antibodies (Table 1) were then applied and left to incubate overnight in a humid chamber at 4°C. This included antibodies to insulin, proinsulin (no cross-reactivity with insulin), glucagon, and DAPI. The following day, the sections underwent a gentle rinse with three cycles of 5-min washing in PBS on a shaker. Subsequently, they were incubated at room temperature in a dark, humid chamber with secondary antibodies for 1.5 h. Afterward, the slides were washed in PBS for three sets of 5-min washes, followed by nuclei staining using 1 uL of a 5 mg/ml stock solution of DAPI mixed with 5 ml PBS. The sections were incubated in DAPI in the dark for 10 min at room temperature. Following this, the slides were washed in PBS for three sets of 5-min washes and then rinsed in ddH_2_O for 5 min. The slides were left to dry in the dark overnight. Finally, a few drops of FluorSave (345789–20 ml) from (EMD Millipore Corp, USA), were placed in the middle of the tissue. A coverslip was positioned, excess mounting media aspirated, the slide sealed, and then again stored in the dark at 4°C.

**Table 1.** List of antibodies utilized for immunofluorescence staining experiments, including their sources, catalog numbers, and dilutions, are provided in the following inventory.

Target	Company	Item Number	Dilution
**Primary Antibodies**			
Insulin	Agilent	IR00261–2	IF: 1:4
Glucagon	Abcam	GR3392735–3	IF: 1:200
Proinsulin	R&D Systems	MAB13361	IF: 1:50
DAPI	MP Biomedicals	157574	IF: 1:5000
**Secondary Antibodies**			
AlexaFluor 568 Donkey anti-mouse	Abcam	ab175472	IF: 1:250
AlexaFluor 488 Donkey anti-Rabbit	Abcam	ab150073	IF: 1:250
AlexaFluor 647 Donkey anti-Guinea Pig	Jackson ImmunoResearch	706-605-148	IF: 1:250

### Microscopy

Images were captured using a Leica TCS SP8 confocal/2P microscope (Leica Microsystems, Inc., Buffalo Grove, IL) at the Indiana Center for Biological Microscopy located at Indiana University, School of Medicine. The imaging process employed a Leica HC PL APO CS2 20×/0.75IMM objective lens adjusted for oil immersion. Scanning was performed in sequential laser illumination mode with three sequences. The first sequence involved photomultiplier tubes (PMT)1 using 405 nm excitation and detecting emissions within the range of 415–483 nm, and PMT4 using 638 nm excitation and detecting emissions within the range of 648–750 nm. The second sequence employed PMT2 with 488 nm excitation and detected emissions within the range of 500–500 nm. Finally, the third sequence utilized PMT3 with 552 nm excitation and detected emissions within the range of 560–635 nm. The acquired images were in a 12-bit grayscale format and had a resolution of 1024 × 1024 pixels (554 × 554 um). The scanning speed was set at 200 hz with 1 AU, bidirectional × scan, and line averaging two resolutions. The hardware scanning setup was consistent for all samples.

### Image quantification and analysis

The quantification of relative intensity for insulin, glucagon, and proinsulin was conducted using ImageJ distribution package software, FIJI (NIH, Bethesda, MD)^7^. The fluorescence channels were analyzed individually, and regions of interest (ROIs) were defined around each islet to determine the islet area, and the same ROIs were applied across the different fluorescence channels. The quantification method for staining each hormone in each islet was determined by applying a threshold value to the pre-defined ROI in the islet. The “max entropy” function was utilized, and the threshold value was adjusted manually and consistently across all images to ensure that only the staining related to the specific antibody was selected. The selected area was then measured, and the ROI was saved for further analysis of fluorescent intensity. To facilitate comparisons between islets of varying sizes, the relative area of the target antigen within each islet was normalized to the respective islet’s overall area. Mean intensity was measured for each channel, and nuclei were counted using the cell counter function within the ROI. The quantification of the fluorescent intensity of insulin, glucagon, and proinsulin, representing the respective hormone content, was obtained by measuring the average fluorescent intensity within the previously identified ROI that corresponded to the areas stained by the specific antibodies. The measurements then normalized by the islet area, and the intensities were reported relative to Phase I WT ferret islets. This normalization process aimed to improve the comparability of results across different islets.

### Ratio analysis

The ratio analysis was performed by obtaining the fluorescence intensity of proinsulin values to insulin (PI:I) staining and calculating proinsulin to insulin, as well as glucagon-to-insulin ratio (Glu:I) for each cell or region. For each sample during each Phase, the proinsulin intensity was divided by the insulin intensity, and glucagon-to-insulin ratio was calculated similarly^8^.

### Colocalization analysis

Colocalization analysis was conducted for insulin and proinsulin staining using the Just Another Colocalization Plugin (JaCoP), accessible via the open-source software ImageJ, FIJI^7^. The analysis covered 41–195 islets and total of 42 images across Phases I–III, with seven images from each group split into two channels. Assessing how much the localization in the green (insulin) channel overlapped with that in the red (proinsulin) channel. The assessment relied on Manders’ Overlap Coefficients (MOC) to quantify the degree of overlap between two channels, disregarding the linearity of the connection^6^. Additionally, Manders’ Overlap Coefficient (proinsulin in insulin-containing pixels) throughout each individual islet was used as a proxy for the fraction of cells in each islet that are failing to produce mature insulin. Threshold values for channel 1 (Green: Insulin) was set to 44 and 39 for channel 2 (Red: Proinsulin). Furthermore, the colocalization percentage between PI and INS was calculated using Mander’s Colocalization Coefficient (M1). The M1 coefficient reflects the fraction of pixels in the insulin (INS) channel that overlap with those in the proinsulin (PI) channel. To convert this value into a percentage, the M1 coefficient is multiplied by 100, providing the percentage of colocalized pixels between the two channels. A perfect colocalization receives a result of 1, and 0, indicating none^9^.

### Statistical analysis

We performed a statistical analysis using GraphPad Prism 9.4 (GraphPad Software, San Diego, CA, USA). The normality of the data was assessed using the Kolmogorov–Smirnov test, which indicated that the data were not normally distributed. Consequently, the data underwent a log_10_ transformation and were analyzed using parametric tests. A two-way ANOVA, followed by Tukey’s multiple comparison post hoc test, was used to compare the statistical significance between WT and CF sections across three phases. The results are presented as mean values ± SEM, with a *p*-value of less than 0.05 considered statistically significant.

## Results

### Insights into insulin staining intensity changes across phases in cystic fibrosis

**Figure 2.**
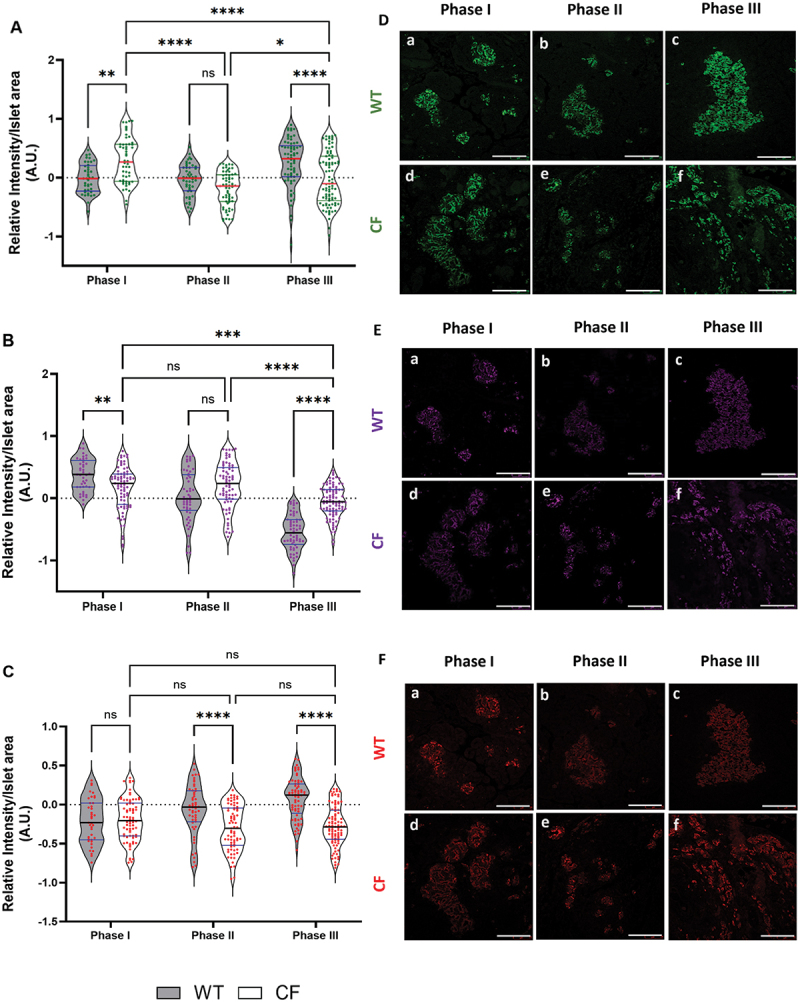
Changes in immunofluorescence staining intensity per islet area assessed in wild-type (WT) ferrets and CFTR knockout ferrets (CF) using ImageJ. A – C: comparisons of relative intensity across phases I-III were conducted between WT and CF ferrets, along with an assessment of CFRD progression within CF ferrets. (A) insulin, (B) glucagon, (C) proinsulin. D – F: Representative images of immunofluorescence staining in Phase I, Phase II, and Phase III for both WT and CF ferrets (D) insulin, (E) glucagon, (F) proinsulin. Scale bar = 75 µm. The analysis included 150–245 islets from 2–4 biological replicates for each genotype in each phase. The data were transformed using log_10_ and analyzed with parametric tests. Two-way ANOVA and Tukey’s multiple comparisons post hoc test was employed to assess statistical significance, and the results are presented as mean-values ± SEM, and significance level is denoted by asterisks with (****p < 0.0001, ***p < 0.001, **p < 0.01, *p < 0.05).

We conducted immunofluorescence staining analysis to elucidate the underlying changes in beta-cells of CF vs. WT ferrets. Our main goal was to compare the expression and distribution of insulin in pancreatic tissues between the two genotypes over time and to clarify the previously described phases of this model. As seen in Phase I (Figure 2A), there was a significant increase in insulin staining in the CF sections compared to WT (*p <*0.01). While, in Phase II, there were no notable differences in insulin intensity between the two groups, however, the mean insulin intensity staining of CF sections decreased despite the test not being statistically significant (*p =* 0.2206). Interestingly, in Phase III group the CF exhibited significantly lower insulin staining compared to the WT group (*p* < 0.0001).

Next, we focused on investigating how insulin staining changed across all three Phases within the CF group. To explore this further, we compared Phase I with Phase II and observed a significant decrease in Phase II relative to Phase I (*p* < 0.0001) and Phase III (*p* < 0.05). Similarly, Phase III demonstrated a significant decrease when compared to Phase I (*p* < 0.0001). In (Figure 2D) Representative images of immunofluorescence staining for insulin are shown for WT and CF across Phases I-III. The reduced insulin staining found in CF indicates a decrease in function that impacts insulin production.

### Glucagon staining in cystic fibrosis in disease progression

Subsequently, we performed the same analysis for glucagon staining. Here, in (Figure 2B) we found a significant difference in the relative intensity of glucagon staining between CF and WT sections during Phase I. Notably, the CF sections displayed significantly decreased glucagon staining compared to the WT group (*p <*0.01), as determined by the intensity per islet area. However, during Phase II there was no significant difference observed between CF and WT although the mean intensity staining of CF sections was higher than WT (*p* = 0.0519). In contrast, Phase III displayed higher intensity glucagon staining for CF (*p <*0.0001).

We then examined the disease progression within the CF group throughout all three phases. We did not observe a significant difference in glucagon intensity staining between Phase I and Phase II. However, comparing Phase III of the CF model to the other Phases, revealed a significant decrease in glucagon intensity during Phase III and Phase I (*p <*0.0001; *p <*0.001) respectively. Representative images of immunofluorescence staining for glucagon are displayed for WT and CF across Phases I-III (Figure 2E).

### Exploring proinsulin staining in cystic fibrosis

To investigate possible insulin processing disorders due to beta-cell dysfunction secondary to impaired cleavage or misfolding^10^, we conducted a relative intensity analysis of proinsulin staining. Our results showed no significant difference in proinsulin relative intensity in the CF compared to the WT sections during Phase I (Figure 2C). Interestingly, in Phase II and Phase III (*p <*0.0001) there was a significant decline in proinsulin staining intensities, coinciding with CFRD progression. In contrast, no significant changes were observed within the CF group across all three phases. Although the analysis of the CF group did not reveal a statistically significant difference, a decrease was observed. However, Phase II decreased 1.54-fold approximately (40.5%) comparing to Phase III. Additionally, Phase III was lower roughly 1.46-fold or (40.6%) than Phase I. Meanwhile, Phase III decreased by about 0.91-folds or (8.7%) compared to Phase I. Representative images of immunofluorescence staining for glucagon are displayed for WT and CF across Phases I-III (Figure 2F). The findings might suggest that a reduction in proinsulin intensity, caused by endoplasmic reticulum (ER) stress due to prolonged hyperglycemia, occurred in the Phase II that continued to decreased proinsulin levels, and challenges in converting it to mature insulin^11^.

### Assessing beta-cell health using the proinsulin-to-insulin ratio as an indicator of dysfunction

**Figure 3.**
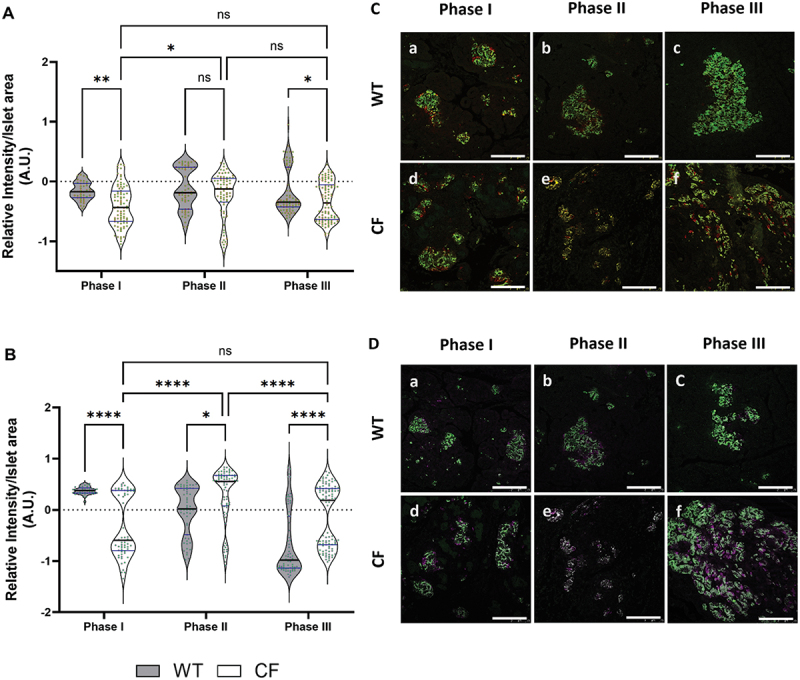
Panel (A) presents violin plot graphs comparing the proinsulin-to-insulin ratio (PI:I). Panel (B) displays violin plot graphs of glucagon-to-insulin ratio (Glu:I) comparisons across phases I-III between CF and WT, along with an assessment of CFRD progression within CF ferrets. In panels (C) and (D) feature representative images of immunofluorescence staining for PI:I and Glu:I in pancreatic tissue of WT and CF ferrets during three different phases. Panel (C) shows an overlay of PI:I channels, with insulin (green) and proinsulin (red). In panel (D) is the overlay channels of Glu:I where with insulin (green) and glucagon (Magenta). Scale bar = 75 µm. The analysis for PI:I included 133–259 islets, while Glu:I contained 68–94 islets from 2–4 biological replicates for each genotype in each phase. The data was transformed using log_10_ analyzed using a two-way ANOVA followed by Tukey’s multiple comparisons post hoc test, with results presented as mean values ± SEM. Significance levels were indicated as follows: (****p < 0.0001, **p < 0.01, and *p < 0.5).

To delve deeper into the condition of beta-cell health, we performed an assessment of the proinsulin-to-insulin ratio (PI:I) as a marker for beta-cell stress^12,13^. This ratio may indicate impaired insulin processing, disturbed insulin secretion, potential beta-cell dysfunction, and disease progression. Our findings revealed a significant decrease in the PI:I in the CF group (*p <*0.01) when compared to the WT ferret sections (Figure 3A). However, there were no significant differences observed in the PI:I for the CF and WT sections in Phase II, but in Phase III, the CF group showed significantly decreased PI:I (*p* < 0.5) as opposed to WT. In contrast, within CF group comparison, Phase II displayed a notable increase in the PI:I compared to Phase I (*p* < 0.5). Phase II showed no significant difference compared to Phase III, nor did Phase I from Phase III.

Representative immunofluorescence staining images for PI:I display for both WT and CF across Phases I-III, with the proinsulin (red) channel merged with the insulin (green) channel (Figure 3C). The reduced PI:I in the CF group compared to the WT group may indicate that the CF group is in a near-normoglycemic stage, where islet regeneration is occurring, or that newly formed islets in the beta-cells are struggling to convert proinsulin into insulin, a condition often associated with beta-cell stress or dysfunction. On the other hand, the lack of a significant difference between Phase II and Phase III, even with a slightly lower mean in Phase III, suggests that any improvement or stabilization in the CF group over time is minimal.

### Evaluating beta-cell function in CFRD using the glucagon-to-insulin ratio

In CFRD, there is a progressive decline in beta-cell function which leads to insulin deficiency, and the degree of this deficiency in relation to glucagon level, provides an insight into the function of the remaining beta-cells. Given the ongoing loss of beta-cell function in CFRD, we evaluated the glucagon-to-insulin ratio (Glu:I)^14,15^. Given the lack of serum samples to measure levels, we used immunofluorescence staining to assess and analyze the ratio of glucagon-producing to insulin-producing cells. In Phase I, we observed a significant decline in Glu:I in CF when compared to the WT sections (*p <*0.0001; Figure 3B). Conversely, the ratio increased in CF sections during Phase II (*p <*0.05) and Phase III (*p <*0.0001). Within the CF group comparison, Phase II sections showed a significant increase in Gl:I than Phase I and Phase III (*p <*0.0001). Meanwhile, no significant difference was detected between Phase I and Phase III. Representative immunofluorescence staining images for Glu:I are shown for both WT and CF across Phases I-III, with the glucagon (magenta) channel merged with the insulin (green) channel (Figure 3D). The elevated Glu:I may suggest worsening insulin insufficiency and/or a deterioration in beta-cell function, both of which are crucial markers of CFRD progression in Phase II and III^4^.

### Proinsulin-to-insulin conversion and beta-cell health: a co-localization perspective

**Figure 4.**
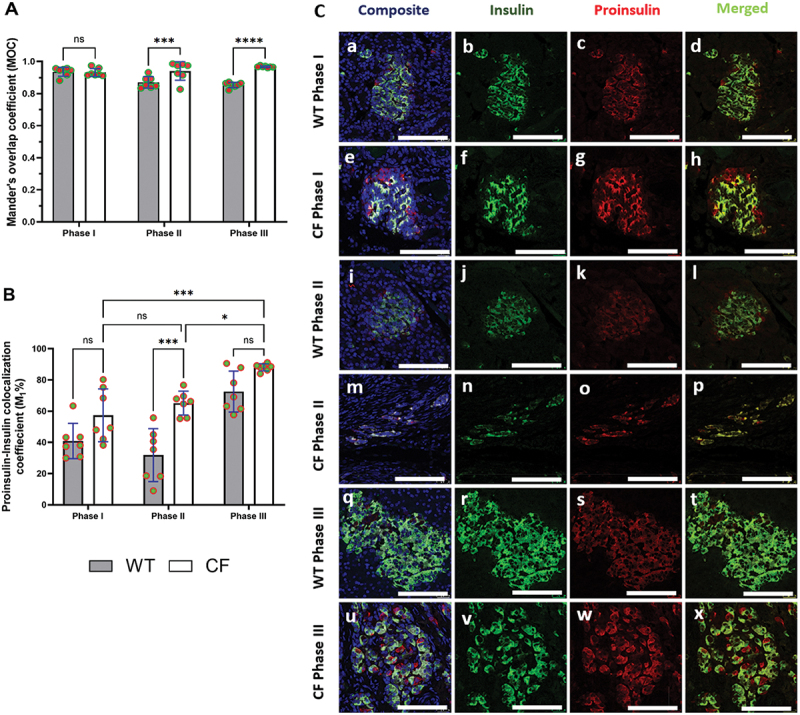
(A) histogram displaying the distribution of the Mander’s overlap coefficient (MOC) of insulin/proinsulin co-localization. (B) illustrating the rate of proinsulin-to-insulin colocalization (M1%). Immunofluorescence images for each genotype across phases I-III, focusing on islets within beta cells in pancreatic sections (C). The composite panels display DAPI (nuclei, blue), green panels (insulin), red panels (proinsulin), and merged panels showing overlay or co-localized channels. The scale bar is 25 μm. The data were normally distributed, so parametric tests, including two-way ANOVA followed by Tukey’s multiple comparisons post hoc test, were used to assess statistical significance, results presented as mean values ± SEM. Significance levels indicated as follows: (****p < 0.0001, ***p < 0.001, and *p < 0.05).

In order to assess the efficiency of proinsulin conversion into mature insulin and to gain comprehensive insights into beta-cell functionality, we conducted a proinsulin-to-insulin co-localization analysis^9^ The results showed that MOC fraction of insulin that colocalized with proinsulin gradually increased in the CF group (Figure 4A). In Phase I, there was no significant difference observed in the insulin over proinsulin overlap between WT and CF groups. However, the overlap coefficient significantly increased in CF during Phase II (*p* < 0.001) and Phase III (*p* < 0.0001) compared to WT.

We then calculated the colocalization percentage rate (Figure 4B), by using proinsulin-insulin colocalization rate (M1) representing the fraction of the first channel’s signal insulin (green) that overlaps with the second channel proinsulin (red)^9^. The results revealed no significant difference between WT and CF in Phase I. In Phase II, CF exhibited a significant increase (*p* < 0.001) compared to WT. Interestingly, Phase III did not show a significant difference from WT, although the increase was 15% higher than WT. On the other hand, we examined the colocalization percentage within the CF group, finding no significant difference between CF and WT in Phases I and II. Notably, Phase III was significantly higher than Phase II (*p* < 0.5) and Phase I (*p* < 0.001).

In the representative immunofluorescence images for proinsulin-to-insulin colocalization analysis (Figure 4C), it is evident that the insulin signal is largely distributed compared to proinsulin in the beta-cells of WT sections, with proinsulin showing partial colocalization with insulin (Figure 3C, panels d, l, and t). Conversely, in the beta-cells of CF, proinsulin displays a distinct distribution and demonstrates a positive colocalization with insulin (Figure 4C, panels h, p, and x). Overall, these results suggest that beta-cells are having difficulty converting proinsulin into mature insulin. This difficulty suggests an increased insulin demand in CF and will ultimately lead to beta-cell dysfunction.

### Quantifying islet shape and size alterations across phases of endocrine dysfunction

In CFRD, pancreatic islets experience functional and structural alterations associated with both endocrine and exocrine dysfunctions^2,16^. To better understand these changes, we conducted measurements using metrics like islet area, circularity, and Feret’s diameter. This analysis provides insights into how CF affects islet architecture and function compared to age-matched WT ferrets^16^.

**Figure 5.**
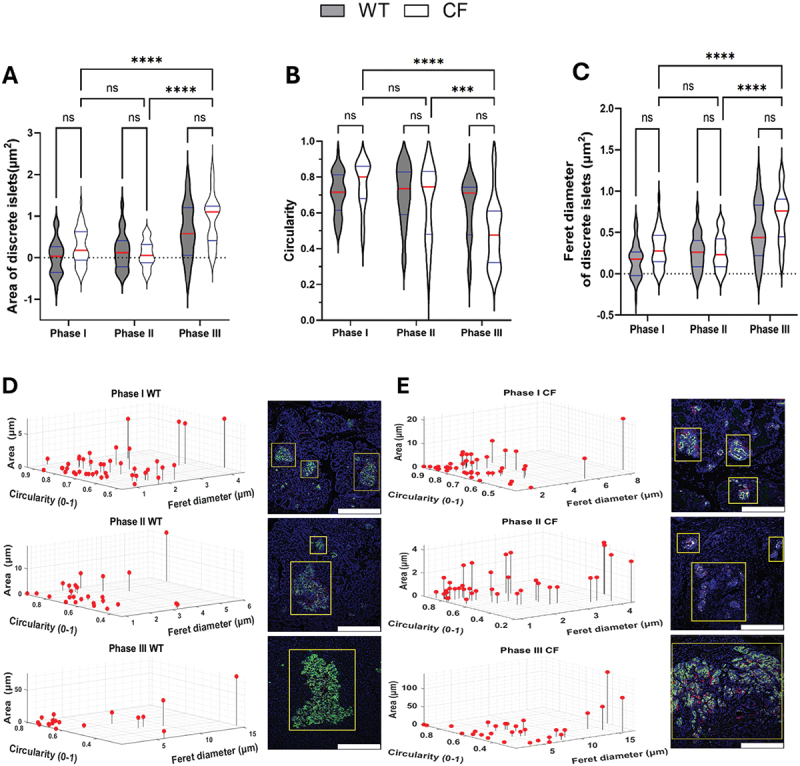
The shape and distribution of islets in both WT and CF ferrets were analyzed to highlight differences between the two groups. This analysis involved assessing key parameters of spatial organization within the pancreas. **A-C**: violin plot graphs showing quantification of islet area (**A**), circularity (**B**), and Feret’s diameter (**C**) for each genotype across Phases I–III. **D** and **E**: three-dimensional visualization of the distribution of islet size (area) and shape (circularity and Feret’s diameter) for WT (**D**) and CF (**E**) ferrets, computed with a custom MATLAB script. Each point corresponds to an individual islet or cluster and the regions identified as islets are marked by yellow boxes in the immunofluorescence images, and the scale bar is 75 μm. The analysis included 41–195 islets, from 2 to 4 biological replicates for each genotype in each phase. The data in panels **B**, **D**, and **E** were not transformed to a logarithmic scale to facilitate easier comparison of the actual values. In contrast, the data in panels A and C were log_10_-transformed and analyzed using a two-way ANOVA followed by Tukey’s multiple comparisons post hoc test, with results presented as mean values ± SEM. Significance levels were indicated as follows: (****p < 0.0001 and ***p < 0.001).

The analysis of the islet area revealed no significant differences between CF and WT sections across all three phases (Figure 5A). However, within the CF group, while there was no significant change in islet size between Phases I and II, a significant increase in islet size was observed in Phase III compared to Phases I and II (*p* < 0.0001).

Next, we performed a circularity analysis to evaluate how closely the shape of the pancreatic islets look like a circle and how this shape changes over time in CF and WT throughout various phases of disease progression. Circularity was quantified on a scale from 0.0 to 1.0, with 1.0 indicating a perfect circle. The result revealed no significant differences found between CF and WT sections across all three phases (Figure 5B). Within the CF group, there was no significant change in circularity between Phases I and II; however, a significant increase in islet size was noted in Phase III compared to both Phases I (*p* < 0.0001) and II (*p* < 0.001).

In addition to measuring area and circularity, we assessed Feret’s diameter to achieve a thorough understanding of islet morphology in CF and WT ferrets (Figure 5C). The Feret’s diameter is the longest diameter of the islet, and it provides valuable insights into the maximum size of the islets which helps to elucidate how the changes in islet structure relate to disease progression and their potential effects on endocrine function. Consistent with the earlier findings, no significant differences were found between CF and WT across all three phases. However, within the CF group, there was a significant increase in Phase III compared to both Phases I and II (*p* < 0.0001).

Furthermore, we generated a three-dimensional scatter plot for the data (Figure 5, D and E), visualizing the correlation between islet size and shape (circularity and Feret’s diameter) distribution by using Custom MATLAB scripts^16,17^. These measurements were not normalized to the total pancreatic area, allowing us to focus on changes in the size of individual islets. In WT sections, the islet area and Feret’s diameter increased with age, while the islets became less circular; and smaller islets tended to retain a more circular shape (Figure 5D). Whereas, in CF sections, we observed that the islet area also increased by approximately 1.44-fold (about 44%, *p* = 0.5126), and ferret diameter increased by 1.4-fold (about 37%) compared to WT. However, circularity decreased by 20% in CF ferrets compared to WT (Figure 5E). Overall, the findings may imply that while both WT and CF experience growth in islet size, however, CF ferrets show greater shape irregularities. This may indicate that disease-related processes in CF are impacting the structure of pancreatic islets.

## Discussion

In this study, we present the results of immunofluorescence staining conducted across the first three phases of glycemia in CF ferrets relative to age-matched WT ferrets. During Phase I of the study, we noticed CF islets exhibited significantly higher levels of insulin expression compared to WT. An increase in the relative intensity of glucagon staining was observed along with reduced proinsulin staining during Phases II and III in CF islets compared to age-matched WT islets.

There was a notably lower PI:I in Phases I and III in the CF group compared to WT. Conversely, there was a significantly higher Glu:I in Phase II and III in CF sections when compared to WT. Mander’s overlap coefficient analysis revealed a significant increase in insulin colocalization with proinsulin in Phases II and III in CF compared to age-matched WT sections. Additionally, analysis of the colocalization percentage rate revealed a significant increase in the proinsulin-insulin colocalization rate (M1) during Phase II in CF sections compared to WT.

The increased intensity of insulin staining in CF compared to WT ferrets in Phase I is intriguing as it may indicate an early response or adaptation of the beta-cells in CF ferrets, possibly in response to physiological stress or other factors related to the disease^5^. In contrast, during Phase II, there were no significant differences in insulin expression between the CF and WT islets, although it trended toward lower expression in CF, which may suggest a decline in pancreatic beta-cell function, reduced insulin secretion, and/or impaired glucose metabolism during the development of diabetes^18,19^. While in Phase III, insulin expression in the CF group once again significantly reduced when comparing to WT. Overall, it appears there are dynamic changes in insulin expression throughout the CFRD stages, with Phase III standing out as a period of particularly distinct patterns of decreased insulin expression in the CF islets^20^. This may indicate a reduction in insulin expression in response to normoglycemia during Phase III, however, without circulating insulin levels, we cannot confirm this. On the other hand, and within the CF sections during the three phases, the decrease in relative insulin expression in Phases II and III compared to Phase I suggests a progressive deterioration in pancreatic beta-cell function over time. As CFRD advances, it gradually impacts pancreatic health, leading to a decrease in insulin production and secretion^20^.

The increased relative intensity of glucagon expression in CF ferrets is consistent with the findings reported by Yi et al.^2^, where they identified elevated glucagon mRNA levels in CF ferret islets. It is well-known that increased glucagon levels, or hyperglucagonemia, are commonly found in both types of diabetes, including type 1 diabetes (T1D) with insulin deficiency and type 2 diabetes (T2D) with hyperinsulinemia, and is observed in animal models and humans^21^. Some researchers argue that excessive glucagon expression may play a more crucial role in diabetes development than insulin deficiency^22,23^, as accumulation of glucagon could potentially play a more important role in the progression of diabetes compared to insufficient insulin production^24^. Interestingly, humans with clinical CFRD often display glucagon deficiency, although the changes in glucagon prior to CFRD development remain insufficiently described^23^. Our results show that differences in glucagon expression between the CF group and WT remain consistent throughout the study, with Phase III exhibiting the most significant disparity. These variations hold significance for comprehending the role of glucagon in CF, and may lead to further investigation of the underlying mechanisms and potential therapeutic strategies for regulating glucagon in CF^25,26^.

This study is, to our knowledge, the first to investigate proinsulin staining in both CF and WT ferrets. In CF ferret sections, we observed a significant decrease in both the PI: I in Phases II and III. This finding was unexpected because one would assume higher proinsulin levels in the CF group during periods of beta-cell stress^27^. However, it is possible that this observed decrease is a result of increased efficiency in proinsulin processing, possibly accompanied by a compensatory upregulation of insulin expression^28^. In contrast to our findings, the elevated PI:I ratio in previous T2D studies indicate a potential loss of beta-cell mass during these phases in CF ferrets^29,30^. Brusco and colleagues discovered that prolonged elevation in beta-cell activity, characterized by increased insulin demand and resistance, triggers ER stress and disrupts the beta-cell’s ability to secrete insulin, this is evidenced by changes in the PI:I within the pancreas of individuals with T2D^9^. Additionally, it has been demonstrated that prolonged overexpression of proinsulin mutants prone to misfolding or elevated stress signals in beta-cells leads to impaired insulin secretion, elevated markers of ER stress, and ultimately, a decline in beta-cell function^31^. This is accompanied by a gradual rise in abnormal proinsulin expression and its intracellular distribution^32^. However, no previous studies have explored the relationship between the proinsulin-to-insulin ratio and insulin sensitivity and secretion in the context of CFRD. Thus, we believe that the significant decrease in the PI:I observed in our CF group indicates that these ferrets shifted from hyperglycemia in Phase II to near-normoglycemic and/or partial recovery stage during Phase III, or may have undergone changes in beta-cell function, possibly adapting to insulin-level demand or trying to compensate for insulin resistance^9,33^. Further research is necessary to explore the underlying causes and implications of this ratio difference.

The interaction between insulin and glucagon is essential for regulating blood glucose levels effectively^34^. Our study represents the first research effort to investigate the ratio of glucagon-to-insulin Glu:I using immunofluorescence techniques in CFRD. The objective of Glu:I analysis was to understand the interaction between alpha-cells and beta-cells and their roles in glucose regulation^35^. Although we did not have access to serum levels, when circulating glucagon levels are elevated, it leads to increased blood glucose levels, and a higher Glu:I suggests either an increased glucagon response and/or reduced insulin response^36^. Studies have shown that serum Glu:I may hold promise in early diabetes diagnosis in pancreatic cancer^14^. Jin et al.^37^, found a significant correlation between the Glu:I and HbA1c levels in individuals with pancreatic cancer, similar to findings observed in T2D studies^38^. Furthermore, it has been shown that high Glu:I impacts glycemic control and elevated ratio may hinder lipid metabolism, possibly promoting fatty liver disease^36,39^. The increased Glu:I observed in our findings might suggest exacerbation of the imbalances between glucagon and insulin producing cells resulting from selective loss of beta-cell mass, and as beta-cells become overstressed, they may transdifferentiate into alpha-cells, potentially increasing the relative mass of alpha-cells^8^. Amo-Shiinoki et al.^15^, reported that beta-cell differentiation happens early in the disease progression in individuals with T2D. Moreover, the notable increase in the alpha-cells-to-beta-cells ratio per islet is strongly associated with the increase of the alpha-cells-to-islet ratio, which eventually resulted in the expansion of the islet area. Therefore, their findings indicate that islet remodeling through dedifferentiation is likely the root cause of beta-cell failure during diabetes progression in human^15^. This may also provide insight into the pathophysiological mechanisms behind the trans-differentiation into alpha-cells observed in CFRD^14,19^.

Under normal conditions of insulin production, one would find relatively little proinsulin in beta-cells, whereas if insulin production is disrupted, one would expect to see an accumulation of proinsulin in beta-cells. We anticipate that this increase would result in a higher proportion of insulin-positive beta-cells showing detectable levels of proinsulin. To test the hypothesis that CF is linked to impaired proinsulin-to-insulin conversion, we performed quantitative colocalization analyses on insulin and proinsulin, which confirmed a significant relationship between the two.

In this context, the MOC fraction of insulin colocalizing with proinsulin significantly increased in the CF group during Phases II and III. The elevated MOC indicates that a greater portion of the insulin signal overlaps with the proinsulin signal, suggesting a disruption in the normal processing of proinsulin to insulin in CF, leading to the accumulation of both molecules within the beta-cells. It is also possible that this is the result of Beta-cell aging as a similar change was seen in the age-matched WT sections, but to a lesser degree.

Furthermore, Brusco et al.^9^, reported a significant proinsulin-insulin colocalization rate (M1) in their T2D study, indicating that impaired beta-cell function is associated with increased insulin demand stemming from the elevated workload on beta-cells, which is characterized by protein misfolding and accumulation. This increased workload ultimately leads to endoplasmic reticulum (ER) stress^9^. Similarly, our results suggest a defect in proinsulin synthesis and/or processing that disrupts insulin maturation and/or trafficking, likely caused by ER stress or other cellular dysfunctions associated with CF during the later phases^9,40^.

The absence of notable differences in islet structure and morphometric analyses between WT and CF ferrets aligns with the results presented by Rotti et al.^16^ reported that aging in WT ferrets leads to larger but less circular pancreatic islets, while CF ferrets show more pronounced irregularities and sparse islet formation in later phases. This finding suggests that WT ferrets undergo compensatory changes in islet shape as they age, whereas the morphology of CF ferrets is likely impacted by disease-related factors, such as chronic inflammation. Notably, the improved glucose tolerance observed in CF ferrets during Phase III, despite their irregular islet structure, raises important questions about the compensatory mechanisms involved and the potential adaptive responses in glucose metabolism^16^. In our findings, we also observed that both WT and CF ferrets show an increase in islet size, suggesting a common physiological response. However, the greater shape irregularities noted in CF ferrets indicate a disease-related effect linked to the development and progression of CFRD.

These irregularities may suggest underlying disease mechanisms that compromise the structural integrity of the pancreatic islets (Figure 2D, panel f), which could hinder their effectiveness in insulin secretion compared to WT (Figure 2A). Furthermore, as CFRD advances to Phase IV, the exocrine pancreas undergoes trans-differentiation into adipose tissue^16^.

It is evident that immunofluorescence staining serves as a valuable tool in investigating nuanced cell variations, assessing protein/gene expression in plasma/serum and tissue/cell homogenates, crucial for understanding cellular heterogeneity^12^.

The limitation of this study is the small sample size, as well as the insufficient availability of serum, adequate tissues, and sections, which restricted our ability to concentrate specifically on immunofluorescence staining. Future studies should consider assessing these levels in conjunction with staining by examining their circulating levels and parallel analysis of the expression and/or activity of processing enzymes. Another potential limitation is translating the findings to human studies, given that the ferret model may not precisely reflect human pathophysiology and disease dynamics. However, access to human islets for staining is increasingly challenging in this patient population because of limited sample availability and especially during the early stages of CFRD development.

Strengths of this study include different analytical approaches and the valuable insights into the progression of pancreatic dysfunction in CF ferrets, characterized by reduced insulin staining and possible compensatory increased glucagon staining. These findings enhance our comprehension of diabetes development in the context of CF and could have implications for potential therapeutic approaches targeting insulin and glucagon pathways. The significance of colocalization analysis in our study provides valuable information about the regulatory mechanisms controlling the processing, trafficking, and secretion of proinsulin and insulin within the cell. This understanding deepens our knowledge of cellular biology related to insulin synthesis and secretion. To fully understand the molecular mechanisms driving these changes and to explore potential interventions for mitigating diabetes impact in individuals with CF, further research is warranted.

In conclusion, our findings suggest significant differences during the various phases of disease progression to CFRD. This research could pave the way for novel treatment strategies aimed at ameliorating pancreatic dysfunction in CFRD at earlier stages of disease.

## Prior presentation

Selected components of this research were summarized in an abstract presented at the 81st Scientific Sessions (2021) held from June 25 to 29, 2021, in a virtual meeting format. Additionally, selected findings were shared via a poster presentation at the Center for Diabetes and Metabolic Diseases, Indiana University School of Medicine, Indianapolis, IN.
